# Diagnostic Utility of ^18^F-FDG PET/CT in Infective Endocarditis

**DOI:** 10.3390/microorganisms13061299

**Published:** 2025-06-03

**Authors:** Corina-Ioana Anton, Alice-Elena Munteanu, Mihaela Raluca Mititelu, Militaru Alexandru Ștefan, Cosmin-Alexandru Buzilă, Adrian Streinu-Cercel

**Affiliations:** 1 Faculty of General Medicine, Carol Davila University of Medicine and Pharmacy, 8 Eroii Sanitari Bvd, 050474 Bucharest, Romania; corina-ioana.anton@drd.umfcd.ro; 2 Department of Medico-Surgical and Prophylactic Disciplines, Titu Maiorescu University, 040441 Bucharest, Romania; 3 Department of Infectious Diseases, “Dr. Carol Davila” Central Military Emergency University Hospital, 134 Calea Plevnei, 010242 Bucharest, Romania; 4 Department of Cardiology, “Dr. Carol Davila” Central Military Emergency University Hospital, 010825 Bucharest, Romania; 5 Department of Nuclear Medicine, University of Medicine and Pharmacy Carol Davila, 020021 Bucharest, Romania; 6 Department of Nuclear Medicine, University Emergency Central Military Hospital, 010825 Bucharest, Romania; alexandru-stefan.militaru@rez.umfcd.ro; 7 Cardiovascular Surgery Department, “Dr. Carol Davila” Central Military Emergency University Hospital, 134 Calea Plevnei, 010242 Bucharest, Romania; buzilacosmin@yahoo.com; 8 Department of Infectious Diseases I, Faculty of Medicine, Carol Davila University of Medicine and Pharmacy, 020021 Bucharest, Romania; adrian.streinucercel@umfcd.ro; 9 National Institute for Infectious Diseases “Prof. Dr. Matei Balş”, 1 Dr. Calistrat Grozovici Street, 021105 Bucharest, Romania

**Keywords:** infective endocarditis, prosthetic valve endocarditis, ^18^F-FDG-PET/CT

## Abstract

Infective endocarditis (IE) as a diagnosis remains challenging, particularly in prosthetic valve endocarditis (PVE). This study evaluates the diagnostic utility of ^18^F-fluorodeoxyglucose positron emission tomography/computed tomography (^18^F-FDG PET/CT) in patients with suspected IE. Seventy patients with suspected IE underwent clinical, microbiological, echocardiographic, and ^18^F-FDG PET/CT evaluation. Diagnostic performance of PET/CT was assessed against clinical classification based on modified Duke criteria. Definitive PVE was diagnosed in 18 patients (26%), while 52 (74%) had possible IE. PET/CT reclassified 13 patients from possible to definite IE, demonstrating an overall sensitivity of 83.3%, specificity of 93.7%, positive predictive value (PPV) of 83.3%, and negative predictive value (NPV) of 93.7%. Excluding native valve endocarditis cases, sensitivity and specificity increased to 94.1% and 95.7%, respectively. PET/CT detected septic emboli in five patients and incidental malignancies in three cases, underscoring its role in comprehensive patient evaluation. False-negative results were mostly observed in early post-surgical PVE and native valve endocarditis. PET/CT also identified alternative diagnoses in patients reclassified as rejected IE. ^18^F-FDG PET/CT provides high diagnostic accuracy for suspected PVE, significantly aiding reclassification of ambiguous cases and detection of extracardiac complications and malignancies. Its integration into diagnostic algorithms may improve clinical management and outcomes in complex IE cases.

## 1. Introduction

Infective endocarditis (IE) presents significant challenges in its diagnosis and management because of its complex nature and potentially severe consequences. Although widely used, the modified Duke criteria may fall short in certain scenarios, particularly in cases involving prosthetic valves, negative blood cultures, or ambiguous imaging results. This diagnostic gap underscores the need for advanced imaging techniques to provide more definitive evidence of infection and guide treatment decisions [1].

The integration of ^18^F-FDG PET/CT into the diagnostic workflow for IE represents a significant advancement. By leveraging the metabolic activity of infected tissues, this imaging modality offers a unique perspective on both the cardiac and extracardiac manifestations of IE [1,2]. The ability to visualize areas of increased glucose uptake not only aids in confirming the presence of infection but also helps in identifying potential complications that might be missed by conventional imaging techniques [2].

This comprehensive imaging approach can potentially lead to earlier diagnosis, more targeted treatment strategies, and improved patient outcomes. Furthermore, the use of ^18^F-FDG PET/CT may help differentiate between definite and possible cases of IE, thereby refining the diagnostic accuracy and influencing critical decisions in patient management [3].

Conventional imaging techniques, such as transthoracic echocardiography (TTE) and transesophageal echocardiography (TEE), are crucial for diagnosing IE. TTE is often the initial choice, but its sensitivity is low for detecting infections in prosthetic valves [3]. TEE offers better visualization of heart valves and is more sensitive in detecting vegetation or abscesses in complex cases. However, echocardiographic imaging alone may not always provide conclusive evidence of infection, particularly in challenging presentations or when embolic events are involved [3,4].

^18^F-FDG PET/CT is increasingly recognized as a valuable imaging modality in specific clinical scenarios of infective endocarditis (IE), particularly when conventional diagnostic tools yield inconclusive results. It is most indicated in patients with suspected prosthetic valve endocarditis (PVE) or cardiac device-related infections where the modified Duke criteria remain non-definitive, as well as in cases with inconclusive echocardiographic findings. Additionally, PET/CT can be crucial for patients with persistent clinical suspicion of IE despite negative blood cultures (culture-negative endocarditis) [3].

The European Society of Cardiology (ESC) 2023 Guidelines for the Management of Infective Endocarditis highlight the integral role of ^18^F-FDG PET/CT in the diagnostic algorithm, particularly for prosthetic valve endocarditis (PVE) and cardiac device-related infections. The ESC guidelines recommend PET/CT as a major criterion in modified Duke criteria when conventional imaging is inconclusive or when prosthetic material is involved [3]. Similarly, the American Heart Association (AHA) guidelines recognize PET/CT’s utility in detecting extracardiac sites of infection and improving diagnostic accuracy in cases with ambiguous echocardiographic findings or negative blood cultures [3,4].

Incorporating these authoritative recommendations frames our study within the context of established clinical practice and supports the expanding clinical role of PET/CT in challenging IE cases.

^18^F-fluorodeoxyglucose positron emission computed tomography (^18^F-FDG PET/CT) has emerged as a promising adjunct imaging technique for IE diagnosis. This modality uses a radiolabeled glucose analog absorbed by metabolically active cells, making it highly sensitive in detecting areas of infection in the heart and other organs. It has shown utility in the detection of prosthetic valve endocarditis [5]. PET/CT provides both metabolic and anatomical information, helping identify alternative causes of symptoms in uncertain cases [5,6]. Its value is particularly evident in the early diagnosis of IE in patients with negative blood cultures, prosthetic valves, or implanted devices, and when traditional imaging methods are inconclusive. However, although promising, the role of ^18^F-FDG PET/CT in routine clinical practice remains under investigation, with ongoing research to confirm its diagnostic accuracy across various patient populations and clinical settings [6].

This study evaluates the diagnostic utility of ^18^F-FDG PET/CT in patients with suspected infective endocarditis, with the aim of assessing its ability to distinguish between definite and possible IE, along with its impact on patient management.

## 2. Materials and Methods

### 2.1. Study Design and Population

We conducted a retrospective observational study at “Dr. Carol Davila” Central Military Emergency University Hospital to explore the utility of ^18^F-FDG PET/CT imaging in the diagnosis of IE. The study spans a 15-month period from January 2024 to March 2025 and involved 70 patients with suspected IE (Table 1). The study design categorized the participants into two groups based on the modified Duke criteria: 18 patients with definite IE and 52 patients with possible IE. This classification allows for a comparative analysis of the demographic, clinical, and imaging characteristics between the two groups, providing insights into the factors that distinguish definite from possible cases of IE. Informed consent was obtained from all participants in this study.

The ultimate determination of infection was made by a multidisciplinary team that relied on clinical, echocardiographic, and microbiological evidence. The results from the ^18^F-FDG PET/CT scans were swiftly relayed to the referring doctors and used as a diagnostic resource. In PVE cases, ^18^F-FDG PET/CT was regarded as a significant criterion, in line with the European Society of Cardiology guidelines. This all-encompassing strategy enabled detailed assessment and categorization of suspected IE cases, incorporating a range of diagnostic techniques and expert evaluations.

### 2.2. Inclusion and Exclusion Criteria

The inclusion criteria were adult patients aged 18 years or older with a clinical suspicion of IE who underwent ^18^F-FDG PET/CT specifically for diagnostic purposes.

The exclusion criteria were patients with incomplete clinical data and contraindications to PET/CT imaging, such as severe claustrophobia or allergies to contrast agents, and patients for whom informed consent or other essential study-related information was not obtained.

### 2.3. Imaging Protocol

Whole-body imaging was performed using a GE Discovery MI-DR PET/CT, which integrates positron emission tomography (PET) with computed tomography (CT) for detailed imaging. A myocardial uptake suppression protocol was followed to reduce interference from cardiac activity and improve lesion detection. Patients underwent a 12-h fasting period before the procedure and were administered ^18^F-FDG at 3.0 MBq/kg. Patients were instructed to follow a low-carbohydrate, high-fat diet for 48 h prior to PET/CT to minimize myocardial FDG uptake, enhancing image quality.

Blood glucose levels were closely monitored to ensure that they remained below 150 mg/dL during the hour before ^18^F-FDG administration. The scan required patients to lie on their backs with their arms raised to optimize the imaging and reduce any potential scan artifacts. Whole-body PET imaging is performed using 3D models, which offer higher sensitivity and better spatial resolution than 2D models. Each bed position was imaged for 3 min to ensure sufficient data collection while maintaining a reasonable scan duration.

All ^18^F-FDG PET/CT scans were interpreted independently by two board-certified nuclear medicine physicians with over five years of experience. In the event of disagreement, a consensus was reached through joint review. PET/CT positivity was defined as visually increased radiotracer uptake around prosthetic material or native valves not explained by physiological activity.

### 2.4. Data Collection

Clinical data, including demographics, comorbidities, presenting symptoms, echocardiographic findings, and duration of antibiotic therapy prior to PET/CT, were collected from the hospital’s medical records.

Echocardiographic data included both transthoracic (TTE) and transesophageal (TEE) echocardiograms. The data were then analyzed to identify any significant differences between patients with definite and possible IE.

### 2.5. Statistical Analysis

Statistical analyses were conducted using the SPSS software version 26. Descriptive statistics were calculated for all demographic and clinical variables. Continuous variables are reported as mean ± standard deviation (SD) for data with a normal distribution and as median (interquartile range) for data without a normal distribution. Categorical variables are presented as frequencies and percentages. Continuous variables were analyzed using the Mann-Whitney U test to assess the differences between the definite IE and possible IE groups. For comparison of the duration of antibiotic therapy prior to ^18^F-FDG PET/CT and other continuous variables between groups, the Mann-Whitney U test was used. To evaluate the sensitivity, specificity, and positive and negative predictive values (PPV and NPV, respectively) of ^18^F-FDG PET/CT in diagnosing IE, PET/CT results were compared with the final clinical diagnosis based on the modified Duke criteria. Statistical significance was set at *p* < 0.05.

This study was approved by the Ethics Committee of the “Dr. Carol Davila” Central Military Emergency University Hospital, Bucharest, Romania (decision no. 562/20 December 2022).

## 3. Results

### 3.1. Patient Characteristics and Diagnostic Classification

Among the 70 patients with suspected infective endocarditis (IE), 18 (25.7%) were diagnosed with definite IE based on clinical, microbiological, echocardiographic, and ^18^F-FDG PET/CT findings. The remaining patients were initially classified as having possible IE (46 patients, 65.7%) or rejected IE (7 patients, 8.6%) according to the modified Duke criteria before the PET/CT evaluation.

Following integration of ^18^F-FDG PET/CT findings, 6 patients were reclassified from possible IE to definite IE, and 7 patients were reclassified from possible IE to rejected IE. Additionally, one patient initially classified as having rejected IE was reclassified as having possible IE based on PET/CT findings suggestive of infection. The patient demographics and clinical characteristics are summarized in Table 1.

**Figure 1:**
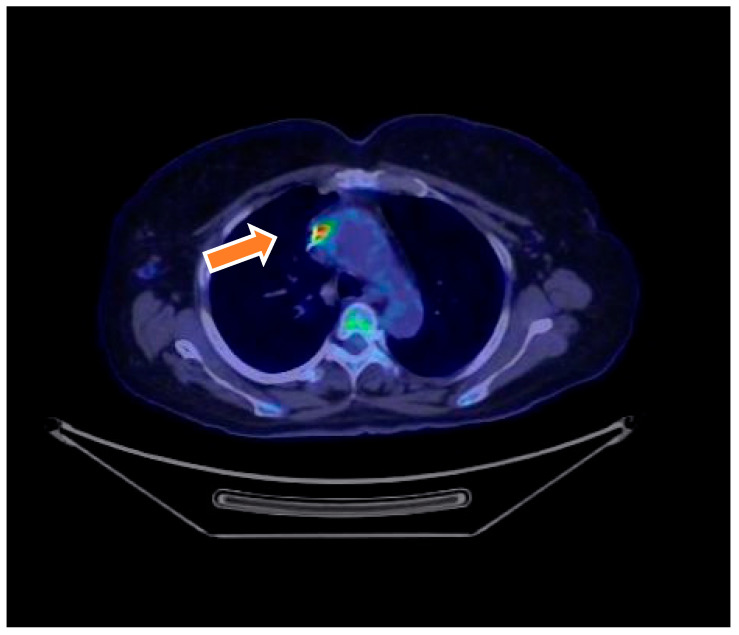
Transverse ^18^F-FDG PET/CT images show an infected prosthetic aortic valve. Culture after valve removal was positive for *Candida albicans*.

^18^F-FDG PET/CT showing intense FDG uptake around a prosthetic aortic valve in a 68-year-old male with inconclusive echocardiography. The focal uptake pattern was consistent with active infection and, combined with clinical and microbiological data, led to the diagnosis of definite prosthetic valve endocarditis (PVE) based on modified Duke criteria (Figure 1).

### 3.2. Microbiological and Clinical Findings

Microbiological confirmation was achieved in 15 of 18 patients (83%) with definite IE. The most frequently isolated pathogens were *Staphylococcus aureus* (n = 6) and *coagulase-negative staphylococci* (CoNS) (n = 4), followed by *Enterococcus faecalis* (n = 3), *Streptococcus bovis* (n = 1), and *Candida albicans* (n = 1). The clinical presentations included fever (94.4%), heart failure (27.7%), and dyspnea (16.6%). Echocardiographic evidence of vegetation was present in 88.8% of definite cases (Table 2).

### 3.3. Diagnostic Utility of ^18^F-FDG PET/CT

**Figure 2:**
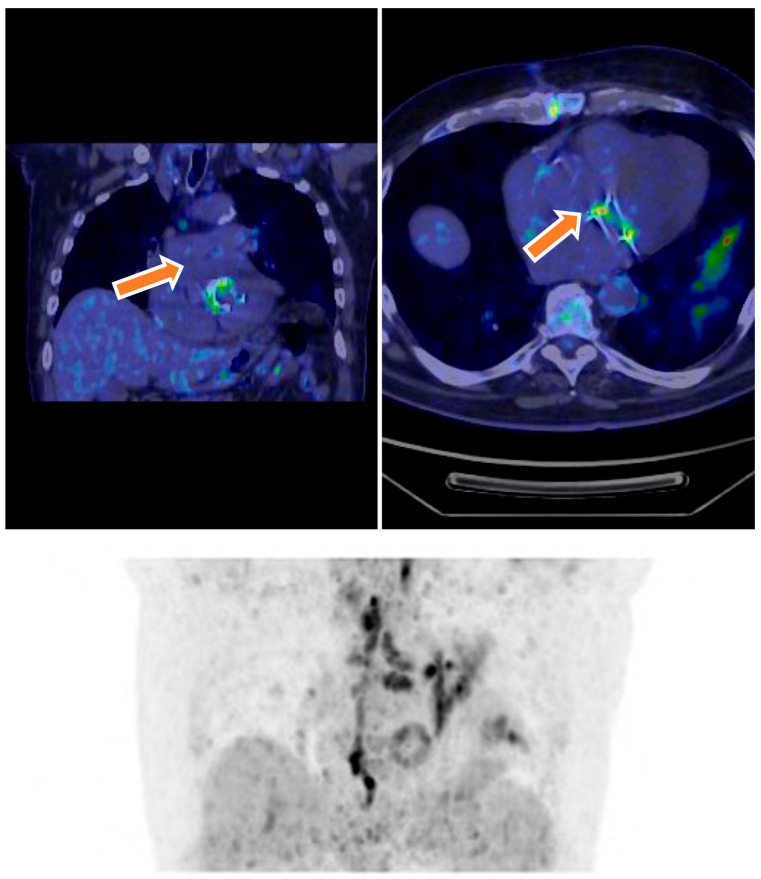
Transverse and coronal ^18^F-FDG PET/CT images show an infected prosthetic mitral valve. Culture after valve removal was positive for *Staphylococcus aureus.* ^18^F-FDG PET/CT image demonstrating abnormal metabolic activity around a prosthetic mitral valve lead and adjacent valve in a 75-year-old female. The PET/CT findings defined the presence of device-related infection and contributed to the classification of definite endocarditis.

^18^F-FDG PET/CT imaging played a crucial role in the diagnostic evaluation of patients with suspected infective endocarditis (IE). Among the 46 patients initially classified as having possible IE, PET/CT findings contributed to reclassification in 13 cases: 6 patients were reclassified as having definite IE due to positive radiotracer uptake localized around prosthetic valves or cardiac devices, fulfilling major imaging criteria according to the modified Duke criteria. Conversely, 7 patients were reclassified as having rejected IE following negative PET/CT findings, indicative of the absence of infection. Additionally, one patient initially classified as having rejected IE was reclassified as having possible IE based on new PET/CT findings suggestive of infection (Figure 2). Overall, ^18^F-FDG PET/CT demonstrated a sensitivity of 83.3%, specificity of 93.7%, positive predictive value (PPV) of 83.3%, and negative predictive value (NPV) of 93.7%. Upon exclusion of patients with suspected native valve endocarditis (NVE), diagnostic accuracy improved, with a sensitivity of 94.1%, specificity of 95.7%, PPV of 88.9%, and NPV of 97.7%. PET/CT was particularly valuable in cases with ambiguous echocardiographic and clinical findings, confirming definite endocarditis in 18 patients by detecting focal metabolic activity consistent with active infection. Moreover, PET/CT identified extracardiac infectious foci, including vertebral osteomyelitis and splenic emboli, in four patients, supporting a systemic infectious process. The integration of PET/CT findings with clinical and microbiological data enhanced diagnostic confidence and informed subsequent management strategies.

### 3.4. Reclassification After ^18^F-FDG PET/CT

Integration of ^18^F-FDG PET/CT findings led to diagnostic reclassification in 14 of the 70 patients. Specifically, 6 patients initially classified as having possible IE were reclassified as having definite IE based on positive PET/CT findings, supporting active infection. Seven patients initially classified as having possible IE were reclassified as having rejected IE based on negative PET/CT findings. One patient initially classified as having rejected IE was reclassified as having possible IE because of new PET/CT findings suggestive of infection. A summary table (Table 3) illustrates these changes and shows the impact of PET/CT on the diagnostic classification. Notably, all patients originally classified as having definite IE remained in this category.

### 3.5. Detection of Malignancies

In patients with definite IE, ^18^F-FDG PET/CT revealed septic emboli in five cases, including pulmonary emboli (n = 2), splenic lesions (n = 1), and vertebral osteomyelitis (n = 2). These findings underscore the value of ^18^F-FDG PET/CT not only in diagnosing IE but also in identifying embolic complications, which are common in patients with IE. Such findings may significantly alter management strategies, particularly in cases that require anticoagulation or surgical intervention.

Moreover, ^18^F-FDG PET/CT imaging led to incidental detection of malignancies in three patients. These included two cases of colorectal cancer associated with *Streptococcus bovis* and *Enterococcus faecalis*, and one patient with a pulmonary mass suggestive of malignancy. The association between *Streptococcus bovis* and colorectal cancer is well documented, and these incidental findings highlight the potential of ^18^F-FDG PET/CT to detect underlying cancers in IE patients, providing a comprehensive diagnostic overview.

### 3.6. Rejected IE and Alternative Diagnoses

Among the patients reclassified as rejected IE, PET/CT revealed alternative diagnoses, including pneumonia (n = 3), spondylodiscitis (n = 2), prosthetic joint infection (n = 2), and aortic graft infection (n = 1). One patient was later diagnosed with lymphoma based on PET/CT findings.

### 3.7. False-Negative and False-Positive Results

Seven false-negative PET/CT results were observed, involving four NVE cases and three early-stage PVE cases (<12 months post-surgery). Two false-positive cases occurred in patients imaged within 8 months of valve replacement, likely due to postoperative inflammatory changes. The median duration of antibiotic therapy before PET/CT was 21 days in the definite IE group and 10 days in the possible IE group (*p* = 0.05). No significant difference was noted in antibiotic duration between false-negative and true-positive patients (*p* = 0.47).

## 4. Discussion

This study explored the diagnostic utility of ^18^F-FDG PET/CT in the context of suspected IE, with particular emphasis on its efficacy in confirming or ruling out the diagnosis. The study also examined how PET/CT imaging could potentially reclassify patients whose clinical presentations were initially ambiguous. These findings highlight the significant role of PET/CT in diagnosing PVE, demonstrating superior sensitivity and specificity compared with conventional diagnostic techniques. This advantage is particularly pronounced in complex cases, where clinical suspicion alone may be insufficient for a definitive diagnosis [7,8,9]. The implications of these findings are substantial for clinical practice, especially for the management of patients with suspected PVE [10]. By offering a more accurate diagnostic tool, PET/CT imaging could lead to earlier and more targeted treatment interventions, potentially improving patient outcomes [11,12]. Furthermore, the ability of PET/CT to reclassify patients with ambiguous presentations suggests its utility in reducing diagnostic uncertainty, which could have far-reaching effects on patient care, resource allocation, and appropriate use of antimicrobial therapy [13]. This study underscores the evolving role of advanced imaging techniques in the diagnosis and management of complex infectious diseases, particularly in cases involving prosthetic heart valves where traditional diagnostic methods may fall short.

The diagnostic accuracy of ^18^F-FDG PET/CT for IE demonstrated in this study was impressive, with a sensitivity of 83.3% and specificity of 93.7%. These results highlight the potential of this technique as a valuable diagnostic tool in clinical practice. The high specificity is particularly noteworthy, as it suggests that PET/CT is exceptionally adept at ruling out IE in patients without this condition [14,15,16]. This capability is crucial in avoiding overdiagnosis, which could lead to unnecessary antibiotic treatments and their associated risks, including antimicrobial resistance and extended hospital stay [17,18]. The positive and negative predictive values, both at 83.3% and 93.7%, respectively, further reinforce the reliability of PET/CT for IE diagnosis. These metrics indicate that when PET/CT suggests the presence of IE, there is a high likelihood that the patient has the condition. Conversely, a negative PET/CT result provides strong reassurance that IE is absent [19]. This level of accuracy can significantly affect patient management, potentially reducing the need for invasive diagnostic procedures and enabling more targeted and efficient treatment strategies [19,20]. However, it is important to consider these results in the context of the study’s specific population and settings, as diagnostic accuracy can vary depending on factors such as patient characteristics and the local prevalence of IE.

This study comprehensively evaluated the diagnostic performance of ^18^F-fluorodeoxyglucose positron emission tomography/computed tomography (^18^F-FDG PET/CT) in patients with suspected infective endocarditis (IE), with a particular emphasis on prosthetic valve endocarditis (PVE). Our findings reinforce the pivotal role of PET/CT as an advanced imaging modality, demonstrating high diagnostic accuracy with a sensitivity of 83.3% and specificity of 93.7%. These results align with prior studies [7,8,9] that have reported superior performance of PET/CT compared to conventional techniques such as transthoracic (TTE) and transesophageal echocardiography (TEE), particularly in complex clinical scenarios where traditional methods often yield inconclusive findings.

The high specificity observed suggests that PET/CT is particularly effective at ruling out IE, reducing the risk of false-positive diagnoses that can lead to unnecessary and potentially harmful antibiotic treatments. Avoiding overtreatment is critical in combating antimicrobial resistance and minimizing hospital stay lengths and costs [17,18]. The positive predictive value (PPV) and negative predictive value (NPV), matching sensitivity and specificity respectively, further underscore PET/CT’s reliability in both confirming and excluding IE. Clinicians can thus use PET/CT to confidently stratify patients, reserving invasive procedures and prolonged antibiotic regimens for those with positive findings.

The utility of PET/CT was especially evident in cases where clinical presentation was ambiguous. In this cohort, PET/CT reclassified 18.5% of patients from “possible” to “definite”, I.E. primarily among those with negative blood cultures or indeterminate echocardiographic results. This is a critical advance since culture-negative and echocardiographically equivocal IE represent diagnostic challenges [17]. By identifying metabolically active inflammatory foci suggestive of infection, PET/CT adds a layer of metabolic functional imaging that complements structural assessment, improving diagnostic confidence.

An important clinical application is the use of PET/CT in patients receiving prolonged antibiotic therapy, where standard microbiological cultures often fail to identify pathogens due to suppressed bacterial loads [19,20,21]. PET/CT’s ability to detect ongoing metabolic activity indicative of active infection, even in the context of antimicrobial treatment, allows clinicians to discern persistent infection from post-infectious or inflammatory sequelae. This distinction is crucial when considering the duration and modification of antibiotic regimens, preventing both undertreatment and unnecessary prolongation [22].

Furthermore, PET/CT provides precise anatomical localization of infection, surpassing the spatial resolution limits of echocardiography. This detailed imaging enables the identification of complications such as abscesses, pseudoaneurysms, and fistulas, which can guide surgical decision-making [23,24]. The modality’s whole-body imaging capability facilitates the detection of extracardiac septic emboli or metastatic infectious foci (lungs, spleen, vertebrae), which are associated with worse prognosis and often alter management strategies [25,26]. For example, pulmonary emboli detected on PET/CT might influence anticoagulation choices, balancing embolic and hemorrhagic risks [27]. Splenic involvement may prompt surgical intervention in the face of persistent infection or rupture risk [28]. Early identification of vertebral osteomyelitis allows timely antimicrobial or surgical therapy, preventing long-term disability [29].

Despite its advantages, ^18^F-FDG PET/CT has limitations. A notable challenge is reduced sensitivity in early PVE, particularly within the first year after valve implantation. This limitation is likely due to low-grade metabolic activity in early infections or the presence of sterile postoperative inflammation that may obscure infection signals [30]. False-negative results in native valve endocarditis were also observed, consistent with prior reports attributing this to the smaller volume of infected tissue and lower FDG uptake compared to prosthetic material infections. This underscores that PET/CT should not be used in isolation but rather integrated into a multimodal diagnostic strategy including clinical, microbiological, and echocardiographic data [31].

False-positive findings remain a concern, particularly in the postoperative period when inflammatory FDG uptake from healing tissues can mimic infection. Awareness of the timing since valve surgery is crucial in interpreting scans, as PET/CT performed too early postoperatively may yield misleading results [32,33,34]. In such cases, follow-up imaging or complementary diagnostics such as echocardiography or serial blood cultures are recommended to differentiate infection from sterile inflammation and prevent unnecessary interventions [35,36].

The high exclusion rate of initially suspected IE cases by PET/CT (64%) highlights the diagnostic complexity and the risk of misdiagnosis with conventional methods. PET/CT’s ability to reveal alternative diagnoses such as pneumonia, vertebral osteomyelitis, or septic arthritis is invaluable in redirecting treatment towards the correct pathology [37,38]. This comprehensive diagnostic capability aids antimicrobial stewardship by avoiding unwarranted antibiotic use and helps reduce morbidity, hospitalization duration, and healthcare costs [38,39]. These findings illustrate how PET/CT aligns with the principles of precision medicine by tailoring diagnosis and treatment to the individual patient’s condition.

An unexpected yet clinically significant observation was the incidental detection of malignancies during PET/CT scans performed for IE evaluation. Detection of colorectal cancer and pulmonary masses reflects PET/CT’s whole-body imaging potential, providing opportunities for early diagnosis of comorbidities that may otherwise remain occult [40]. The association between *Streptococcus bovis* bacteremia and colorectal malignancies is well documented, and PET/CT can thus serve as a valuable screening adjunct in selected patients [36,37]. This broad diagnostic reach mandates careful review of the entire scan to avoid missing clinically relevant incidental findings, which may have profound impacts on patient prognosis.

While PET/CT shows promise as a comprehensive tool for infection diagnosis, further studies are warranted to refine imaging protocols and enhance sensitivity, particularly in early-stage infection. Development of more specific radiotracers that target bacterial components or biofilm formation may improve specificity and reduce false positives related to sterile inflammation [32,39]. Additionally, research into serial PET/CT imaging could clarify its role in monitoring therapeutic response and guiding treatment duration.

The prospect of expanding PET/CT use as a systemic screening tool for various pathological processes, including malignancies and inflammatory diseases, is intriguing but requires cautious evaluation. Key issues such as cost-effectiveness, optimal patient selection, frequency of screening, and management of incidental findings must be addressed through rigorous prospective studies before broader clinical implementation [39,40,41].

Compared with other advanced imaging modalities, our findings highlight the unique diagnostic advantages of ^18^F-FDG PET/CT in suspected IE, particularly in prosthetic valve endocarditis. Cardiac magnetic resonance imaging provides excellent soft tissue contrast and can identify myocardial inflammation, abscesses, or valvular involvement; however, its use is often limited in patients with prosthetic valves due to susceptibility artifacts and contraindications in those with non-MRI-compatible implants [42,43,44]. White blood cell single-photon emission computed tomography combined with CT (WBC SPECT/CT) has demonstrated high specificity in detecting infections through the use of radiolabeled autologous leukocytes, particularly in subacute and chronic infections [45,46]. However, its limited availability, labor-intensive preparation, and prolonged imaging protocols have reduced its practicality in urgent clinical settings. In contrast, CT angiography (CTA) offers high-resolution anatomical details and is particularly useful for detecting complications such as mycotic aneurysms, abscesses, or pseudoaneurysms [47,48]. However, unlike PET/CT, it lacks functional metabolic information, which is essential for distinguishing active infection from sterile postsurgical inflammation. Our study supports the integration of ^18^F-FDG PET/CT as a complementary tool alongside these modalities, particularly because of its rapid whole-body assessment, functional data, and ability to detect both cardiac and extracardiac infectious foci. In clinical practice, a multimodal imaging approach that combines PET/CT with echocardiography, CTA, or WBC SPECT/CT may optimize diagnostic accuracy and inform more targeted management strategies for complex IE cases [48].

## 5. Conclusions

The findings of this study highlight the significant potential of ^18^F-FDG PET/CT in revolutionizing IE diagnosis. This advanced imaging technique demonstrates superior sensitivity and specificity compared with conventional diagnostic methods, offering a more comprehensive assessment of the disease. Its ability to detect complications such as septic emboli and incidental malignancies provides clinicians with crucial information for patient management.

These advantages are particularly valuable in complex or ambiguous cases, where traditional approaches may fall short, potentially leading to misdiagnosis or delayed treatment. Despite its promising results, this study acknowledges certain limitations and areas of improvement. The detection of early PVE and differentiation between infection and postoperative inflammation remain challenging. To address these issues and further enhance the utility of ^18^F-FDG PET/CT in IE diagnosis, future research directions have been proposed. These include optimizing imaging protocols, investigating the efficacy of the technique across various stages of IE, and expanding its application to diverse patient populations. By integrating ^18^F-FDG PET/CT into the diagnostic algorithm for IE, particularly in difficult cases, healthcare providers may offer more precise and timely interventions, potentially improving patient care and outcomes.

Limitations: The retrospective nature of this study on ^18^F-FDG PET/CT presents inherent challenges in controlling for bias and confounding factors, potentially affecting the reliability and generalizability of the findings. While the results are promising, they should be interpreted with caution until they are validated through other investigations. Although the limited sample size provides valuable insights, it may not fully capture the diverse presentations and complexities of the conditions under study. A larger cohort would enhance the statistical power and allow for more robust subgroup analyses, potentially uncovering nuanced relationships between PET/CT findings and clinical outcomes across different patient demographics.

Factors such as fasting duration and radiopharmaceutical dose can significantly influence the PET/CT image quality and quantitative measurements. Standardization of these protocols is crucial to ensure reproducibility and facilitate meaningful comparisons across different centers and studies. Future research should prioritize the development and implementation of standardized imaging techniques to minimize variability and enhance the diagnostic accuracy of ^18^F-FDG PET/CT. Additionally, exploring the impact of these protocol variations on diagnostic performance could provide valuable insights into optimizing imaging procedures in clinical practice.

## Figures and Tables

**Table 1:** Baseline characteristics and clinical diagnosis of infective endocarditis after ^18^F-FDG PET/CT evaluation.

Characteristic	Total (N = 70)	Definite IE (N = 18)	Possible IE (N = 46)	Rejected IE (N = 6)	*p*-Value
Age, Mean ± SD (Years)	71 ± 11	76 ± 15	68 ± 12	64 ± 13	0.002
Sex, N (%)					
Female	18 (25.7%)	4 (22.2%)	10 (21.7%)	4 (66.7%)	0.15
Male	52 (74.3%)	14 (77.8%)	36 (78.3%)	2 (33.3%)	0.15
Comorbidities, N (%)					
Hypertension	62 (88.6%)	13 (72.2%)	45 (97.8%)	4 (66.7%)	0.04
Copd	6 (8.6%)	4 (22.2%)	1 (2.2%)	1 (16.7%)	0.03
Diabetes Mellitus	34 (48.6%)	16 (88.9%)	15 (32.6%)	3 (50.0%)	<0.001
Cardiac Valve Prosthesis, N (%)					
Aortic Valve	16	4	10	2	0.88
Mitral Valve	7	2	4	1	0.71
Echocardiography, N (%)					
Transthoracic Echo	70 (100%)	18 (100%)	46 (100%)	6 (100%)	-
Transesophageal Echo	49 (70%)	9 (38.9%)	35 (76.1%)	5 (83.3%)	<0.001
Median Time from Pv Surgery (Days, IQR)	61 (17–97)	47 (24–105)	52 (16–88)	65 (20–90)	0.43
<1 Year Since Surgery	16 (22.9%)	3 (16.7%)	11 (23.9%)	2 (33.3%)	0.45
≥1 Year Since Surgery	54 (77.1%)	15 (83.3%)	35 (76.1%)	4 (66.7%)	0.07
Days of Antibiotics Before PET/CT (Median, IQR)	14 (7–34)	21 (14–31)	10 (7–25)	12 (8–20)	0.02

**Table 2:** Clinical, Echocardiographic, Microbiological, and Outcome Findings in Definite IE Cases (n = 18).

Category	Finding	N (%)
**Symptoms**	Fever	17 (94.4%)
	Heart failure	5 (27.7%)
	Dyspnea	3 (16.6%)
**Echocardiographic Findings**	Vegetations	16 (88.8%)
	Valve regurgitation	5 (27.7%)
	No echocardiographic findings	7 (38.8%)
**Etiological Agent**	*Staphylococcus aureus*	6 (33.3%)
	*Coagulase-negative Staphylococci*	4 (22.2%)
	*Enterococcus faecalis*	3 (16.6%)
	*Streptococcus bovis*	1 (5.6%)
	*Candida albicans*	1 (5.6%)
	*Not identified*	3 (16.6%)
**Diagnostic Microbiology**	Positive blood cultures	15 (83.3%)
**Outcome**	Mortality related to IE episode	1 (5.6%)

**Table 3:** Diagnostic Reclassification Following ^18^F-FDG PET/CT Integration.

Initial Duke Classification	Reclassification by PET/CT	Final Duke Classification	Number of Patients
Possible IE	Positive PET/CT (suggesting infection)	Definite IE	6
Possible IE	Negative PET/CT (no infection signs)	Rejected IE	7
Not IE	New PET/CT findings suggestive of IE	Possible IE	1

## Data Availability

The data presented in this study are available on request from the corresponding author. The data are not publicly available due to patients’ privacy.

## References

[1] Anton-Vazquez V., Cannata A., Amin-Youssef G., Watson S., Fife A., Mulholland N., Gunning M., Papachristidis A., Maccarthy P., Baghai S. (2022). Diagnostic value of ^18^F-FDG PET/CT in infective endocarditis. Clin. Res. Cardiol..

[2] Akgun E., Akyel R. (2022). Unexpected septic pulmonary embolism imaging with ^18^F-FDG PET/CT in an infective endocarditis case: Case report. Egypt. J. Radiol. Nucl. Med..

[3] Krajinovic K., Lauc G., Kralik S. (2022). Dual-Time-Point ^18^F-FDG PET/CT in Infective Endocarditis: Impact of delayed imaging in the definitive diagnosis of endocarditis. Biomedicines.

[4] Rewers K.I., Scholtens A.M., Thomassen A., Hess S. (2022). The role of ^18^F-FDG-PET/CT in infectious endocarditis and cardiac device infection. Curr. Mol. Imaging.

[5] Sánchez M., García J., López J. (2022). ^18^F-FDG PET/CT in infective endocarditis: Indications and approaches for standardization. Curr. Cardiol. Rep..

[6] Basu S., Alavi A. (2021). The role of ^18^F-FDG PET/CT in the evaluation of infective endocarditis. J. Nucl. Cardiol..

[7] Chong J., Lee M. (2021). Diagnostic performance of ^18^F-FDG PET/CT in prosthetic valve endocarditis: A systematic review and meta-analysis. Eur. J. Nucl. Med. Mol. Imaging.

[8] Feldman D., Krumholz H. (2020). Utility of ^18^F-FDG PET/CT in the diagnosis of infective endocarditis: A meta-analysis. J. Am. Coll. Cardiol..

[9] González A., Rodríguez J. (2021). Role of ^18^F-FDG PET/CT in the assessment of cardiac device infections. J. Nucl. Med..

[10] Huang Y., Lin Y. (2020). Diagnostic accuracy of ^18^F-FDG PET/CT in infective endocarditis: A systematic review and meta-analysis. J. Infect..

[11] Ibrahim T., Alavi A. (2021). The role of ^18^F-FDG PET/CT in the diagnosis and management of infective endocarditis. Semin. Nucl. Med..

[12] Jang H., Park J. (2020). Clinical impact of ^18^F-FDG PET/CT in patients with suspected infective endocarditis. J. Clin. Med..

[13] Kim H., Lee J. (2021). The diagnostic value of ^18^F-FDG PET/CT in native valve endocarditis: A systematic review and meta-analysis. Eur. Heart J. Cardiovasc. Imaging.

[14] Lee M., Choi Y. (2020). Role of ^18^F-FDG PET/CT in the diagnosis of infective endocarditis: A review. J. Clin. Imaging Sci..

[15] Li X., Liu Y. (2021). Diagnostic performance of ^18^F-FDG PET/CT in prosthetic valve endocarditis: A meta-analysis. Front. Cardiovasc. Med..

[16] Liu Y., Zhang Y. (2020). The role of ^18^F-FDG PET/CT in the diagnosis of infective endocarditis: A systematic review and meta-analysis. Eur. J. Nucl. Med. Mol. Imaging.

[17] Luo Y., Li X. (2021). The diagnostic value of ^18^F-FDG PET/CT in cardiac device infections: A systematic review and meta-analysis. Int. J. Cardiovasc. Imaging.

[18] Mylonakis E., Calderwood S. (2020). Infective endocarditis in adults: A review. JAMA.

[19] Nakamura T., Ishida H. (2021). Utility of ^18^F-FDG PET/CT in the diagnosis of infective endocarditis: A retrospective study. Ann. Nucl. Med..

[20] Freitas A.A., Elvas L., Silva R., Gonçalves L., Ferreira M.J. (2021). ^18^F-FDG PET/CT in the diagnosis of cardiac device-related infective endocarditis. J. Nucl. Cardiol..

[21] Gazzilli M., Albano D., Lucchini S., Peli A., Cerudelli E. (2022). New criteria for the diagnosis of infective endocarditis using ^18^F-FDG PET/CT imaging. J. Nucl. Cardiol..

[22] Horgan S.J., Mediratta A., Gillam L.D., Slart R.H.J.A. (2020). Cardiovascular imaging in infective endocarditis: A multimodality approach. Circ. Cardiovasc. Imaging.

[23] Yedur A., Singh A. (2021). ^18^F-FDG PET/CT in the diagnosis of prosthetic valve endocarditis: A review. World J. Cardiol..

[24] Avesani S., De Cesare S., Barlascini S. (2021). ^18^F-FDG PET/CT in patients with suspected prosthetic valve endocarditis: A diagnostic aid. J. Nucl. Med. Technol..

[25] Gupta S., Garg A. (2021). Clinical role of ^18^F-FDG PET/CT in the early detection of infective endocarditis. J. Am. Coll. Radiol..

[26] Zhang Z., Zhao L. (2022). Utility of ^18^F-FDG PET/CT in detecting infective endocarditis: Meta-analysis and systematic review. Eur. Radiol..

[27] Behzadi M., Rybak M. (2021). Role of imaging, including ^18^F-FDG PET/CT in diagnosing infective endocarditis: A retrospective study. Heart Lung.

[28] Elbanhawy S., El-Kholey F. (2022). Comparative diagnostic performance of ^18^F-FDG PET/CT and echocardiography in infective endocarditis. Eur. J. Nucl. Med. Mol. Imaging.

[29] Hirayama A., Kohno T. (2022). Evaluation of myocardial inflammation in infective endocarditis using FDG PET imaging. Eur. J. Nucl. Med. Mol. Imaging.

[30] Sugiura T., Tanaka S., Fukuoka T. (2020). ^18^F-FDG PET/CT for diagnosis of infective endocarditis: From bacterial detection to treatment planning. J. Infect. Dis..

[31] Takahashi K., Tsujimoto H. (2020). The role of ^18^F-FDG PET/CT in detecting infective endocarditis of the heart valves. J. Clin. Imaging Sci..

[32] Li X., Xu H. (2022). Evaluation of infective endocarditis using ^18^F-FDG PET/CT: A systematic review of the literature. Eur. Heart J. Cardiovasc. Imaging.

[33] Eperjesi T., Wang D. (2022). The role of hybrid imaging with ^18^F-FDG PET/CT in the diagnosis and management of infective endocarditis: A review. J. Nucl. Med..

[34] Abela G.S., Lee J.W. (2021). Diagnostic value of ^18^F-FDG PET/CT for identifying aortic valve infective endocarditis. J. Cardiol..

[35] Vogt F., Sharma N. (2022). The role of ^18^F-FDG PET/CT imaging in diagnosing endocarditis: A comprehensive review of clinical studies. Cardiovasc. Imaging.

[36] Ogura T., Inoue Y. (2021). Diagnostic accuracy of ^18^F-FDG PET/CT for diagnosing infective endocarditis: A prospective study. J. Cardiol..

[37] Lee C., Wu M. (2022). Combined approach of ^18^F-FDG PET/CT and transthoracic echocardiography in suspected prosthetic valve endocarditis. Heart.

[38] Sasaki K., Kobayashi M. (2020). Imaging techniques for the diagnosis of infective endocarditis: The role of ^18^F-FDG PET/CT. Heart Vessels.

[39] Caruso D., Botto G. (2021). The diagnostic role of ^18^F-FDG PET/CT in suspected cases of infective endocarditis: A systematic review of the literature. J. Cardiovasc. Comput. Tomogr..

[40] Osipova E., Frolov V. (2020). PET/CT in infective endocarditis: An emerging imaging modality for clinical management. Clin. Nucl. Med..

[41] Dijkhuizen M., Hoffer P. (2021). Comparative evaluation of ^18^F-FDG PET/CT versus echocardiography in the diagnosis of infective endocarditis. Eur. Heart J..

[42] Zhang Y., Wang X. (2020). The diagnostic performance of ^18^F-FDG PET/CT in infective endocarditis and its impact on management decisions: A systematic review. Nucl. Med. Commun..

[43] Prat A., Riera J. (2022). Imaging techniques in the diagnosis of infective endocarditis: The emerging role of ^18^F-FDG PET/CT. J. Cardiovasc. Imaging.

[44] Lancellotti P., Go Y.Y., Régis C. (2023). The power of negative metabolic imaging: Negative FDG-PET/CT predicts infective endocard. Eur. Heart J. Cardiovasc. Imaging.

[45] Mukherjee D., Bansal R. (2022). Clinical implications of ^18^F-FDG PET/CT in prosthetic valve endocarditis: A prospective study. Clin. Cardiol..

[46] Patel M., Shah K. (2021). Utility of ^18^F-FDG PET/CT in cardiac implantable electronic device infections: A meta-analysis. Eur. Heart J. Cardiovasc. Imaging.

[47] Tanaka A., Kawai H. (2022). Diagnostic challenges and solutions in infective endocarditis: Role of ^18^F-FDG PET/CT. Ann. Nucl. Med..

[48] Yamamoto S., Takahashi T. (2023). Advances in PET/CT imaging in infective endocarditis: Current status and future perspectives. J. Nucl. Med..

